# *Pichia stipitis* genomics, transcriptomics, and gene clusters

**DOI:** 10.1111/j.1567-1364.2009.00525.x

**Published:** 2009-07-29

**Authors:** Thomas W Jeffries, Jennifer R Headman Van Vleet

**Affiliations:** 1 USDA Forest Products LaboratoryMadison, WI, USA; 2 Department of Bacteriology, University of Wisconsin-MadisonMadison, WI, USA

**Keywords:** yeast, evolution, genome, tandem repeats, orthologs, expression arrays

## Abstract

Genome sequencing and subsequent global gene expression studies have advanced our understanding of the lignocellulose-fermenting yeast *Pichia stipitis*. These studies have provided an insight into its central carbon metabolism, and analysis of its genome has revealed numerous functional gene clusters and tandem repeats. Specialized physiological traits are often the result of several gene products acting together. When coinheritance is necessary for the overall physiological function, recombination and selection favor colocation of these genes in a cluster. These are particularly evident in strongly conserved and idiomatic traits. In some cases, the functional clusters consist of multiple gene families. Phylogenetic analyses of the members in each family show that once formed, functional clusters undergo duplication and differentiation. Genome-wide expression analysis reveals that regulatory patterns of clusters are similar after they have duplicated and that the expression profiles evolve along with functional differentiation of the clusters. Orthologous gene families appear to arise through tandem gene duplication, followed by differentiation in the regulatory and coding regions of the gene. Genome-wide expression analysis combined with cross-species comparisons of functional gene clusters should reveal many more aspects of eukaryotic physiology.

## Introduction

*Pichia stipitis* has a set of physiological traits that make it very useful for the bioconversion of lignocellulose. In addition to its extensively studied capacity for xylose fermentation, it is also able to ferment, glucose, mannose, galactose and cellobiose along with mannan and xylan oligomers. This makes it a potent organism for hydrolysate or simultaneous saccharification and fermentation (SSF). After glucose, xylose is the second most abundant hemicellulosic component in agricultural residues and fast-growing hardwood species, and cellobiose is the primary sugar formed in enzymatic hydrolysis.

Hemicellulosic sugars are readily recovered following treatment with alkali, dilute acid or autohydrolysis; hence, their use is critical to economical bioconversion. The capacity of *P. stipitis* to use cellobiose is important in SSF because commercial cellulase preparations are often deficient in β-glucosidase and the accumulation of cellobiose inhibits cellulase activities. The capacity of *P. stipitis* to use oligomeric sugars is important because mild acid pretreatments can avoid the formation of sugar degradation products, but can leave 15–50% of the solubilized hemicellulose in an oligomeric form.

Hemicellulosic sugars are underutilized even though they can be readily recovered with higher yields and at lower cost than glucose from cellulose. While they can be converted into a number of useful products such as xylitol, lactic acid and other chemicals, the product with the largest potential market is ethanol. Production of ethanol from lignocellulosic sources will continue to increase as a consequence of energy and agriculture policy incentives to create renewable alternative fuels and reduce carbon dioxide emissions.

Researchers in numerous laboratories have borrowed genes from *P. stipitis* and other fermentative microorganisms to modify *Saccharomyces cerevisiae* for xylose, xylan or cellulose metabolism. While partly successful, efficient xylose utilization has been impaired by *S. cerevisiae*'s generally low rate of xylose consumption and its inappropriate regulatory responses. It lacks sufficient levels of the assimilatory genes, sugar transporters and mechanisms for balancing cofactor levels under oxygen-limiting conditions.

We understand most of the biochemical steps required for xylose utilization, but a more thorough knowledge of how native xylose-fermenting yeasts such as *P. stipitis* regulate their metabolism could advance the field on several fronts. Recent genomic and transcriptomic studies have revealed the presence of functional gene clusters that mediate the use of lignocellulosic substrates. This review briefly examines the physiological and fermentative properties of *P. stipitis*, and focuses on its genomic and regulatory features.

## A novel yeast for lignocellulose bioconversion

Rational metabolic engineering requires a detailed understanding of physiology, biochemistry and genetics. This is possible only when the major pathways and mechanisms are known. Biochemical and genetic characterization of xylose fermentation by *P. stipitis* Pignal (1967) (*Yamadazyma stipitis*) has been underway for at least 15 years since the development of systems for its genetic transformation (Yang *et al.*, 1994; Lu *et al.*, 1998a; Laplaza *et al.*, 2006;) and mating (Melake *et al.*, 1996). Relatively few researchers, however, have attempted its rational modification despite the fact that native strains produce more ethanol from xylose than any other studied yeast – including genetically modified *S. cerevisiae*.

*Pichia stipitis* has the highest native capacity for xylose fermentation of any known microorganism (van Dijken *et al.*, 1986; du Preez *et al.*, 1989;). This yeast was originally isolated from insect larvae and is closely related to several yeast endosymbionts of passalid beetles (Nardi *et al.*, 2006) that inhabit and degrade white-rotted hardwood (Suh *et al.*, 2003). It is a predominantly haploid, homothallic, hemiascomycetous yeast (Vaughan Martini, 1984; Kurtzman, 1990; Gupthar, 1994; Melake *et al.*, 1996;) that forms buds along with pseudomycelia during vegetative growth, and two hat-shaped ascospores from each ascus. Fed batch *P. stipitis* cultures produce up to 47 g L^−1^ of ethanol from xylose at 30 °C (du Preez *et al.*, 1989) with ethanol yields of 0.35–0.44 g g^−1^ xylose (Hahn-Hägerdal & Pamment, 2004), and they are capable of fermenting sugars from hemicellulosic acid hydrolysates with a yield equivalent to about 80% of the maximum theoretical conversion efficiency (Nigam, 2001a,b;).

The genome of *P. stipitis* codes for cellulases, mannases, xylanase and other degradative enzymes that enable survival and growth in a wood-inhabiting, insect-gut environment (Nardi *et al.*, 2006). *Pichia stipitis* has the capacity to ferment xylose, xylan (Lee *et al.*, 1986; Ozcan *et al.*, 1991;) and cellobiose, and to use all of the major sugars found in wood, including arabinose and rhamnose (Koivistoinen *et al.*, 2008). For these reasons, *P. stipitis* has been a common source of genes for engineering xylose metabolism in *S. cerevisiae* (Jeffries & Jin, 2004).

*Pichia stipitis* also has a number of other bioconversion-related traits: it modifies low-molecular-weight lignin moieties (Targonski, 1992), reduces acyclic enones to the corresponding alcohols (Conceicao *et al.*, 2003), forms various esters and aroma components (Fuganti *et al.*, 1993) and can be engineered to produce lactic acid (Ilmen *et al.*, 2007) or xylitol (Kim *et al.*, 2001; Rodrigues *et al.*, 2008;) in high yield. Strains of *P. stipitis* have also been selected for resistance to furfural and hydroxy-methyl furfural (Liu *et al.*, 2005).

Metabolic engineering and adaptive evolution of *S. cerevisiae* for xylose fermentation has been successful to varying degrees (Harhangi *et al.*, 2003; Sonderegger *et al.*, 2004; Karhumaa *et al.*, 2005;). Engineering it with the basic assimilatory machinery of *XYL1, XYL2*, *XYL3* (or *XKS1*), *TAL1, TKL1*, *RPE1* and *RPI1* enables ethanol production. Expressing xylose isomerase (Wiedemann *et al.*, 2006; van Maris *et al.*, 2007; Wiedemann & Boles, 2008;) or xylose reductases and xylitol dehydrogenases with altered cofactor specificities (Matsushika *et al.*, 2008; Petschacher & Nidetzky, 2008;) reduces cofactor imbalances, and increases the ethanol yield. It is not yet clear as to which of these engineering approaches will prove to be more successful in *S. cerevisiae* (Karhumaa *et al.*, 2007).

Overexpression of *P. stipitis* or other fungal sugar transporters can also improve the performance of engineered *S. cerevisiae* on xylose (Weierstall *et al.*, 1999; Saloheimo *et al.*, 2007; Hector *et al.*, 2008; Katahira *et al.*, 2008; Leandro *et al.*, 2008;), but additional regulatory engineering is necessary because *S. cerevisiae* does not possess mechanisms to coordinate ethanol production in response to xylose (Jin *et al.*, 2004). Therefore, even though the genetic tools, detailed biochemical knowledge and physiological properties of *S. cerevisiae* hold great promise for engineering the fermentation of xylose, xylan, cellulose, arabinose, rhamnose (Koivistoinen *et al.*, 2008) and other sugars, much remains to be learned from *P. stipitis* and other yeasts that use these substrates natively. Conversely, the mechanisms *S. cerevisiae* use to ferment xylose can be adapted to improve the performance of *P. stipitis*.

*Pichia stipitis* shunts most of its metabolic flux into ethanol, and produces very little xylitol, but its fermentation rate on xylose is low relative to that of *S. cerevisiae* on glucose. Glucose and xylose are not equivalent fermentations for many reasons, but increasing the capacity of *P. stipitis* for rapid xylose fermentation could greatly improve its usefulness in commercial applications. Unlike *S. cerevisiae*, which regulates fermentation by sensing the presence of glucose, *P. stipitis* induces fermentative activity in response to oxygen limitation (Passoth *et al.*, 1996, 2003a, b; Klinner *et al.*, 2005). This, however, does not constitute the only fermentative regulatory mechanism. Global expression array analysis has shown specific response patterns for xylose, cellobiose, arabinose, rhamnose and other lignocellulosic substrates. It is not fully known whether these are attributable to carbon catabolite derepression or specific induction. Our expression array results show evidence for both.

## Physiological features of *P. stipitis*

Most of the research with *P. stipitis* has focused on its capacity to ferment xylose. Even so, relatively little has been established concerning the rate-limiting steps in ethanol production from this sugar. In an early work, Bicho *et al.* (1988) showed that xylose reductase (Xyl1) and xylitol dehydrogenase (Xyl2) are repressed by glucose and induced during growth on xylose. Xylose is generally not consumed in the presence of glucose; hence, under glucose repression, these activities, along with xylose transport, are rate limiting. In a respiration-limited, *cyc1* mutant of *P. stipitis*, however, xylose is used coincidently with glucose as compared with the *CYC1* parental strain (Shi *et al.*, 1999), suggesting that reducing ATP production can bring about a partial derepression of xylose assimilation.

Increasing the expression of *XYL1* for xylose reductase (Takuma *et al.*, 1991) increased the enzymatic activity almost twofold, but had no beneficial effect on ethanol production (Dahn *et al.*, 1996). To date, the overexpression of *XYL2* in *P. stipitis* has not been examined (Kotter *et al.*, 1990); however, deletion of *XYL2* blocks xylose utilization at the level of xylitol and prevents its growth on this carbon source (Shi *et al.*, 2000; Kim *et al.*, 2001; Laplaza *et al.*, 2006;). d-Xylulokinase activity (Xks1) (Ho *et al.*, 1998) limits the rate of xylose assimilation by *S. cerevisiae* (Richard *et al.*, 2000; Karhumaa *et al.*, 2005;). d-Xylokinase (Xyl3) does not, however, appear to be rate limiting in *P. stipitis* once it is induced on xylose. Xyl3 from *P. stipitis* exhibits about three times the specific activity of Xks1 from *S. cerevisiae*, and cells can still metabolize xylose via a bypass pathway even in a *xyl3*Δ background (Jin *et al.*, 2002). This indicates that a second pentose kinase pathway is active in *P. stipitis*.

Deletion of the *P. stipitis ADH1*and *ADH2* genes (Cho & Jeffries, 1998; Passoth *et al.*, 1998;) decreases ethanol production dramatically, while increasing xylitol production. Adh activities for *P. stipitis* increase under oxygen-limiting conditions (Cho & Jeffries, 1999; Passoth *et al.*, 2003a,b;) in much the same manner as that observed with *Candida shehatae* (Alexander *et al.*, 1987). Pyruvate decarboxylase activities are also induced under oxygen-limiting conditions along with increasing fermentative activity (Lu *et al.*, 1998b; Passoth *et al.*, 1998;). Taken together, these findings suggest that the final steps of the fermentative pathway direct the flow of the reductant from xylitol to ethanol.

The respiratory capacity of *P. stipitis* is notably greater than that of *S. cerevisiae*. Particularly, *P. stipitis* possesses an alternative, nonphosphorylating terminal oxidase (Shi *et al.*, 2002) in addition to a fully functional NADH dehydrogenase complex (respiratory complex I), both of which are lacking in *S. cerevisiae*. While these enable much higher growth yields and the capacity to grow at very low oxygen levels, they also reduce the intracellular NADH supply for fermentation and result in higher cell yields than with *S. cerevisiae*. Deleting *P. stipitis* cytochrome *c* (*CYC1*) reduces the cell yield and growth rate, while shunting more substrate into ethanol (Shi *et al.*, 1999). Deleting the alternative oxidase reduces the capacity of *P. stipitis* to scavenge oxygen at low levels.

*Pichia stipitis* possesses β-xylosidase (Manzanares *et al.*, 1999; Basaran & Ozcan, 2008;) and native Family 11 xylanase activities. The latter has been cloned and characterized from *P. stipitis* NRRL Y-11543 (Basaran *et al.*, 2001). The published xylanases sequence does not match with any identified ORF in the sequenced genome of *P. stipitis* CBS 6054 (=NRRL Y-11545, ATCC 58785), but the sequenced genome does include Family 10 endo-1,4-β-xylanase, and endoglucanase activities that might also act on xylan. *Pichia stipitis*'s native xylanase activity has been supplemented through heterologous expression (Passoth & Hahn-Hagerdal, 2000; Gorgens *et al.*, 2005;).

## Genetic tools

Genetic manipulation of *P. stipitis* is more difficult than with *S. cerevisiae* for multiple reasons. First, there are relatively few useful transformable *P. stipitis* host strains. Second, *P. stipitis* is resistant to most common antibiotics. Third, it uses an alternative codon system that substitutes serine for leucine when a CUG is encountered, which means that expression of foreign proteins, including those used as drug resistance markers, frequently requires some codon modification. Lastly, random (nonhomologous) integration is far more frequent than site-specific integration, which makes targeted deletions much harder to obtain. With these difficulties, however, researchers have made progress in developing genetic tools.

Melake *et al.* (1996) first developed a sporulation and mating system for this yeast; Yang *et al.* (1994) developed a genetic transformation system based on the *ura3* selectable marker; Lu *et al.* (1998a) expanded this to *leu2*; and Piontek *et al.* (1998) developed *trp5* and *his3* as auxotrophic markers in combination with a heterologous autonomous replication sequence. Laplaza *et al.* (2006) developed the first useful drug resistance marker, Sh ble (zeocin), and the loxP/Cre excision system by modifying the CUG codons in these proteins (Sugita & Nakase, 1999). Deletion of the *KU80* gene that is responsible for nonhomologous end joining significantly increases the fraction of homologous recombinant transformants, albeit at the expense of transformation frequency (Maassen *et al.*, 2008). Other yeast-based transformation vectors are also useful with *P. stipitis* (Klabunde *et al.*, 2003). With the publication of the *P. stipitis* genome, interest in and genetic tools for this organism have been steadily growing. A summary of strain development and genetic tools useful for *P. stipitis* has been published recently (Jeffries, 2008).

## The *P. stipitis* genome

With sequencing and annotation of its genome (Jeffries *et al.*, 2007), many diverse elements of the physiology of this yeast have come to light. Space permits only a brief summation of genes that can be found in their most complete annotations on the Joint Genome Institute web site and in other formats on the NCBI, Genamics and KEGG web sites. This publication constitutes a review of those published features. In addition to its original sequence, the SHI-21 *cyc1* mutant of *P. stipitis* CBS 6054 has been resequenced (Smith *et al.*, 2008), revealing a total of 14 point mutations accumulated through mutation and selection over a period of about 7 years.

Sequencing showed that *P. stipitis* CBS 6054 possesses eight chromosomes, of which two pairs are very similar in size, accounting for the earlier results suggesting the presence of six chromosomes (Passoth *et al.*, 1992). Synteny analysis with its nearest completely sequenced yeast genome neighbor, *Debaryomyces hansenii*, showed extensive recombination and shuffling of the chromosomes, which appears to be a common feature.

Sequencing the *P. stipitis* genome has yielded numerous gene targets for metabolic engineering. In addition to the basic pathway for xylose, arabinose, mannose, galactose and rhamnose (Koivistoinen *et al.*, 2008) metabolism, the genome has a number of sugar transporters (Weierstall *et al.*, 1999), β-glucosidases, endoglucanases and a slew of NADH and NADPH-linked alcohol dehydrogenases, all of which could contribute to the fermentative capacities of the host.

## Expression array studies

Genomic sequences alone do not reveal much about the function of a gene unless it is highly conserved and its function is known in other organisms. While the structures of genes coding for many core functions are highly similar to those found in *S. cerevisiae* and other fungi, genes coding for idiomatic functions are notably different. Expression arrays have proved vital in understanding the functions of *P. stipitis* genes. Moreover, multiple homologs often have different expression patterns indicative of different functions.

Consistent culture conditions are very important in obtaining data that can be compared across different experiments. All cultivations for expression arrays described here were performed in New Brunswick Scientific Bioflo 110 3-L bioreactors with working volumes of 2 L each. The bioreactors were equipped with two impellers rotating at 750 r.p.m. The bioreactor temperature was controlled at 30 °C and the pH was kept constant at 5.0 by automatic addition of 5 N KOH. Airflow into the bioreactors for aerobic cultivations was 1 v.v.m. *Pichia stipitis* CBS 6054 was cultivated aerobically as described above and an aerobic transcriptomics sample was obtained during the exponential growth phase. Following sample collection, air and nitrogen were mixed to an initial oxygen concentration of 2% O_2_ using a Matheson gas proportioner, and the bioreactors were sparged at a rate of 0.5 v.v.m. Cultures were grown under low oxygen for 10–12 h, and a second, low-oxygen transcriptomics sample was obtained. Cultivations and sample collections for each carbon source were performed in triplicate.

A defined minimal medium containing trace metal elements and vitamins in the following amounts was used in all bioreactor cultivations: 2.4 g urea L^−1^, 3 g KH_2_PO_4_ L^−1^, 0.5 g MgSO_4_·7H_2_O L^−1^, 1 mL trace element solution L^−1^, 1 mL vitamin solution L^−1^ and 0.05 mL antifoam 289 L^−1^ (Sigma A-8436)(modified from Verduyn *et al.*, 1992). For glucose, xylose or arabinose cultivations, a starting concentration of 50 g L^−1^ of sugar was used. For cellobiose cultivations, a starting concentration of 30 g L^−1^ of cellobiose was used.

Transcriptomics samples were centrifuged for 5 min at 4500 ***g*** in an Eppendorf 5804R centrifuge equipped with an A-4-44 rotor. The supernatant solutions were removed, and the cell pellets were flash frozen in liquid nitrogen. Cell pellets were stored at −80 °C before RNA extraction. RNA was extracted using the Qiagen RNEasy Maxi kit (Qiagen). Cell breakage was accomplished using glass beads in a BeadBeater (Biospec Products) equipped with a 15-mL chamber. RNA was later purified and concentrated further using the Qiagen RNEasy Mini kit (Qiagen). Total RNA was submitted to the University of Wisconsin-Madison Gene Expression Center for cDNA synthesis, and Nimblegen conducted labeling and chip hybridization.

Sixty-mer oligo expression array chips were designed against all of the ORFs recognized in December 2007. Each oligo was present in five internal replicates. Expression array data were analyzed using arraystar 2.0.

## Regulation of pentose phosphate pathway (PPP), glycolysis and tricarboxylic acid (TCA) cycle

**Fig. 1:**
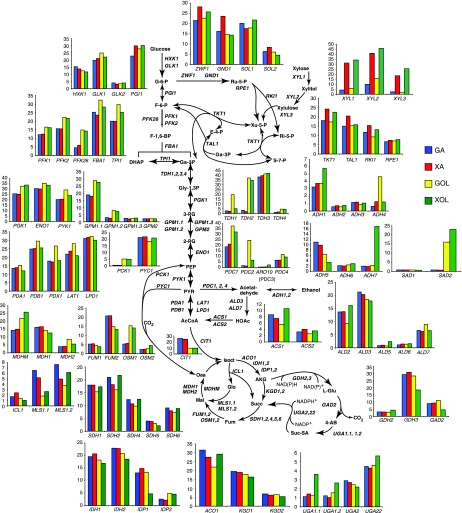
Transcript expression levels of glycolytic enzymes in *Pichia stipitis*. Triplicate cultures were grown at 30°C, pH 5, in 2 L of minimal defined medium with 50 g L^−1^ glucose (G) or xylose (X) under fully aerobic (A) or oxygen-limited (OL) conditions. Cell samples were harvested for mRNA, converted to cDNA and hybridized against 60-mer NimbleGen expression arrays. The average relative expression levels of normalized triplicate samples are shown. Gene designations follow the annotation posted at http://genome.jgi-psf.org/Picst3/Picst3.home.html

In *P. stipitis* and other Crabtree-negative yeasts, fermentation is induced in response to oxygen limitation rather than the presence of glucose (Passoth *et al.*, 1996; Kiers *et al.*, 1998; Cho & Jeffries, 1999;). Previous published studies on fermentation regulation in Crabtree-negative yeasts have apparently not included global transcriptional expression arrays. Describing the complete results would require a book rather than a short review, but Fig. 1 summarizes the expression levels of 88 transcripts of genes thought to be involved directly or indirectly with the PPP, glycolysis and TCA cycle, with cells grown on glucose or xylose under aerobic or oxygen-limited conditions. Note that the vertical axes of these graphs vary with each group of genes in order to show the relative changes of low-abundance transcripts.

Notably, about half of the transcripts do not appear to change significantly under these four cultivation conditions. Second, many of the changes that appear to be significant are relatively small. Third, changes that might be deemed insignificant when seen individually seem more substantive when viewed in the context of other related genes or homologs.

The three genes of the xylose assimilation pathway, *XYL1, XYL2* and *XYL3*, are clearly upregulated on xylose under aerobic or oxygen-limited conditions, and are repressed or downregulated on glucose. Glucose repression of these three genes has been recognized for some time. What is interesting in the context of a global expression analysis is that at its peak, the expression of *XYL2* is among the most abundant of all transcripts in the cell. Data suggest that other transcripts of the PPP are slightly elevated when cells are cultivated on xylose as opposed to glucose. While the changes might seem small, these activities are required under all growth conditions, and the transcript levels are high in all samples; hence, a 20% increase in transcript on xylose under aerobic or oxygen-limiting conditions is significant. Also, the effect is more convincing when similar changes are observed in more than half of the genes simultaneously. By a similar argument, transcripts for the glycolytic enzymes, Pfk1, Pfk2, Fba1, Tpi1, Pgk1, Gpm1.1 and Gpm1.2 all seem to show small increases on glucose, and to a lesser extent, xylose, under fermentative conditions.

Among the most dramatically regulated transcripts are those for triosephosphate dehydrogenase (*TDH1, 2*, *3* and *4*). Whereas *TDH3* is constitutive and among the most abundant of all transcripts, *TDH2* is strongly induced on either xylose or glucose, and *TDH1* is induced on glucose under oxygen limitation. This pattern of one isoform being expressed constitutively with other isoforms for the same activity induced under other conditions – presumably in response to increased metabolic demand – is reflected in *PDC1* and *PDC2, UGA1.1* and *UGA1.2, MSL1.1* and *MSL1.2* as well as other examples not described in this review. This suggests that something limits how much transcript can be produced by a single promoter on a single gene, and that to accommodate the higher levels required for growth, multiple copies evolve with different expression or kinetic patterns.

The abundance of transcripts for *ADH1* and *ADH2* are low relative to the levels of those for other glycolytic or fermentative enzymes and transcripts for the corresponding enzymes found in *S. cerevisiae*. These two activities are essential for ethanol production in *P. stipitis* because deletion of either one or the other significantly reduces ethanol formation, and deletion of both eliminates it almost entirely (Cho & Jeffries, 1998). The induction patterns for *ADH4, ADH7* and *SAD2* on glucose, xylose and under oxygen limitation are not fully understood, in part because their corresponding functions under these conditions are not known.

## Regulatory pathways

Very little is as yet known about regulatory pathways in *P. stipitis*, and what has been gathered from expression array studies requires further confirmation by targeted deletions and physiological studies. With these caveats, however, we can project some possible pathways based on structural similarities and comparative functions in *S. cerevisiae* and other yeasts. Some regulatory proteins are fairly well conserved among *P. stipitis, S. cerevisiae, Candida albicans* and other fungi. The more conserved regulatory proteins such as Gcn1, Gcn2 and Gcn4 show strong one-to-one correlations with corresponding proteins in *S. cerevisiae* (Table 1), and their functions are assumed to be similar. Other proteins – some of which have well-studied functions in *S. cerevisiae*, such as Adr1, Hap1, Mig1 and Gcr1/Gcr2 – have only low (or no) similarity to proteins in *P. stipitis*, or they are similar to multiple proteins so a one-to-one correlation cannot be established. The genes in Table 1 are arranged in a rough descending order based on the degree of conservation observed between *P. stipitis* and *S. cerevisiae*. A comparison between *P. stipitis* and another more closely related yeast such as *C. albicans* would almost certainly show a higher degree of conservation, but because regulation has been best studied in *S. cerevisiae*, that is a standard frame of reference.

**Table 1:** Regulatory genes in *Saccharomyces cerevisiae* with corresponding proteins in *Pichia stipitis*

*S. cerevisiae*	*P. stipitis*	Description of protein function	Comments
*GCN1*	*GCN1*	Translational activator of *GCN4*	Conserved protein
*HAP5*	*HAP5*	CCAAT-binding transcription factor	Conserved protein
*DAL80*	*GZF3*	Negative regulator of genes in multiple nitrogen degradation pathways	Conserved protein
*SNF1*	*SNF1*	AMP-activated serine/threonine protein kinase; required to transcribe glucose-repressed genes	Conserved in *P. stipitis*; 17 similar proteins
*GRR1*	*GRR1*	F-box protein component; involved in carbon catabolite repression, glucose-dependent divalent cation transport, high-affinity glucose transport, morphogenesis and sulfite detoxification	Well conserved in *P. stipitis*; one other gene with slight similarity
*YAP1*	*YAP1/CAP1*	Response to oxidative stress/oxygen detoxification	Moderately conserved
*REB1*	*REB1*	Involved in pol II and pol I transcription, repressor	Moderately conserved
*HAP2*	*HAP2*	Transcriptional activator	Moderately conserved
*GCN4*	*GCN4*	Transcriptional activator of amino acid biosynthesis	Relatively low identity
*HAP4*	*HAP4*	Positive regulator of cytochrome *c* genes	Multiple similar genes
*MIG1*	*MIG1*	Sequence-specific DNA-binding protein involved in glucose repression; regulated by SNF1 kinase and the GLC7 phosphatase	MIG1p has weak identity with nine proteins in *P. stipitis*
*SWI14*	*SWI14*	Mediates cell-cycle-dependent transcription of HO	Some similarity
*SWI16*	*SWI16*	Together with Swi4p, it forms the factor SBF	Similarity
*XBP1*	*XBP1*	Stress-induced repressor	Weak similarity
*NRG1*	*NRG1*	Transcriptional repressor for glucose repression	Similar to *ScNRG1*
*MSM4*	*MSM4*	Regulates carbon source utilization	Similar to several
*HAP1*	*HAP1.1, 1.2*, *1.3*	Fungal transcriptional regulatory protein, similar to *CYP1/HAP1* transcriptional activator	Multiple genes
*HAP3*	*HAP3.1, 3.2*	CCAAT-binding factor, subunit A	Multiple genes
*GAL4*	*GAL4, LAC9*	DNA-binding transcription factor required for the activation of the GAL genes	Similarity to PsLAC9p and PsGAL4p
*MIG2*	*MIG2, MIG2.2*	Transcription factor involved in glucose repression	*PsMIG2.2*, immediately adjacent to *PsGCN1*
*ADR1*	*ADR1.1, ZMS1*	Carbon source-responsive Zn-finger protein	Ten proteins show low similarity to ScADR1p
*GLN3*	*GLN3*	Transcriptional activator of genes regulated by nitrogen catabolite repression	Four similar proteins in *P. stipitis*
*INO2, INO4*	*INO4*	Transcription activator required for derepression of phospholipid biosynthetic genes	Slight similarity in *P. stipitis*
*RGT1*	*–*	Glucose-responsive transcription factor regulating expression of glucose transporters (HXT)	One protein with slight similarity in *P. stipitis*
*GCR1, GCR2*	*–*	Interactive DNA-binding heterodimer transcriptional activators of glycolytic genes	No corresponding proteins in *P. stipitis*

Genes for several proteins particularly play important idiomatic roles in *S. cerevisiae*, but they have no recognizable counterparts in *P. stipitis*. These include the Ino2 transcriptional activator of phospholipids, which could be involved in ethanol tolerance; Rgt1, which is involved in activating glucose transport; and Gcr1/Gcr2, which are transcriptional activators of glycolytic genes. Ethanol tolerance and high glycolytic flux are traits that have evolved as part of *S. cerevisiae*'s fermentative mode of growth and competitive survival in high-sugar environments. Because *S. cerevisiae* represses respiration in the presence of glucose whereas *P. stipitis* induces fermentative activities in response to oxygen limitation, differences in the glucose and oxygen regulatory pathways would be expected. *Pichia stipitis* has evolved in the relatively low-sugar environment prevailing in the beetle mid-gut; hence, its capacity for ethanol tolerance and high glycolytic flux are notably less.

Much of the *S. cerevisiae* pathway for sensing glucose seems to be conserved in *P. stipitis*. Relevant proteins showing high structural similarity include Rgt2, Grr1, Snf1, Sks1, Snf3, Mig2, Mig2.2 and Nrg1. Genes showing little or no conservation include ScMig1/CreA and ScAdr1. Proteins involved in the pathway for oxygen regulation are also conserved in *P. stipitis*. Proteins showing significant similarity include Hap5, Hap3 and Hap2. Proteins with weak or no significant identity in *P. stipitis* include ScHap1 and ScHap4.

The *P. stipitis* genome contains >200 putative Zn-finger regulatory proteins, many of which are poorly conserved or annotated. Because 5′ sequences and regulatory processes appear to evolve much more rapidly than ORFs coding for enzymatic activities, the divergence and diversity of proteins for regulatory responses should not be surprising.

The regulatory pathway that induces fermentative enzymes in *P. stipitis* and other Crabtree-negative yeasts under oxygen-limiting conditions still needs to be resolved. In this regard, transcript levels for *SKS1* and *SNF3* are upregulated about twofold under oxygen-limited conditions on either glucose or xylose. Levels of *GRR1* are essentially unchanged. Specific regulatory responses to glucose and xylose under oxygen limitation are complex, currently under analysis and will be the subject of future publications.

## Functional gene clusters

Functional gene clusters are defined as physically proximal genes sharing common physiological or metabolic attributes. These fall loosely into two groups: (1) pairs or clusters of nonhomologous genes in which each cluster has a single function such as galactose metabolism, and (2) tandem repeats of paralogous genes. Functional clusters are not to be confused with structurally related genes across a range of species that constitute orthologous clusters (Dehal & Boore, 2006). Nor are they to be confused with operons that are prevalent in bacteria, because each gene possesses its own promoter and terminator. Rather, they represent a set of genes coding for proteins with physiologically related functions that confer a survival advantage when coinherited.

The most conspicuous functional clusters in *P. stipitis* consist of two or more families of homologs associated in different ways in different clusters. For the purpose of this review, we define gene families as proteins belonging to orthologous groups as evidenced by common InterPro domain architectures or consistent phylogenetic relationships. Proteins duplicating and diverging within a species are termed paralogs, while proteins having common functions in different species are termed orthologs. In *P. stipitis*, we have evidence that pairs or clusters of proteins having different orthologous origins have duplicated as clusters and diverged into different functions.

In some cases, genes in a functional cluster are coregulated. They often appear in subtelomeric regions where recombination occurs frequently. While gene clusters have been recognized in *S. cerevisiae* and other yeasts, they seem to be particularly abundant in *P. stipitis*. Cross-genomic comparisons show that some clusters for basic functions such as urea metabolism (Table 2) are widely conserved across species, while others for more idiomatic functions seem to be unique. Histones, for example, are present in four heterodimer pairs. These are not tandem duplications of individual genes. Rather, each pair consists of an H3, H4 or H2A H2B subunit, presumably because they function in that manner. This appears to be a highly conserved cross-species cluster. Because clusters are so prominent in *P. stipitis*, a few hypotheses can be drawn from their features.

**Table 2:** Functional gene clusters in *Pichia stipitis*

Cluster name	Genes in cluster	Location	Orientations
β-Glucosidases	*SUT2-BGL1-HXT2.6*	Ch4: 1774-1783 K	Conv., tand.
β-Glucosidases	*HXT2.5-BGL3-SUT3*	Ch6: 1708-1717 K	Tand., conv.
β-Glucosidases	*HXT2.4-EGC2-BGL5*	Ch1: 614-626 K	Div., tand.
β-Glucosidases	*BGL2-HXT2.3-HGT2*	Ch2: 2707.5-2721 K†	Div., tand.
Endo-glucanase	*HXT2.1-EGC3*	Ch1: 2798.5-2803 K	Div.
Endo-glucanase	*BGL6-EGC1*	Ch1: 656.5-662 K	Tand.
α-Glucosidase	*MAL1-MAL6*	Ch2: 752.5-757.2 K	Div.
α-Glucosidase	*MAL2 - MAL7* (*SUC1.1*)	Ch5: 4.5-9.3 K†	Div. (flanking)
α-Glucosidase*	*MAL3-AGL1 YIC1-MAL5* (*SUC1.4, SUC1.2*)	Ch6: 26-42 K†	Div., div. (flanking)
β-Galactosidase	*BMS1-LAC3*	Ch3: 24-250 K	Div.
β-Galactosidase	*LAC1-LAC4*	Ch2: 17812-17869	Div.
Galactose metabolism*	*GAL1-GAL10-*(*unk*)*-GAL102-GAL7*	Ch3: 420-430 K	Div., (tand., conv.), div.
*N*-acetyl glucosamine	*NAG4-NAG2-NAG1-NAG5*	Ch6: 11-19 k†	Conv., div., conv.
l-Rhamnose met	*LRA3-LRA-LRA2-LRA4*	Ch8: 185.8-190.5 K	Tand.
Urea permease	*DUR3.1-DUR1* (*DUR1,2*)	Ch1: 1257. 5-1276 K	Tand.
Iron metabolism	*FTH1-FRE1.2*	Ch2: 1381.5-1386.5 K	Tand.
Iron metabolism	*FRE1.1-FRE1.3*	Ch1: 1318.6-1324.7 K	Tand.
Pyrimidine metabolism	*TPN1-THI4-THI13*	Ch3: 1234-1238.4 K	Div., tand.
Dityrosine formation*	*DIT2-DIT1-DTR1*	Ch2: 1315.9-1321.2 K	Div., tand.
Histone (H3, H4)*	*HHT1-HHF1; HHF1.1-HHT1.1*	Ch6: 620.9-622.8 K Ch6: 106-106.1 K	Div.
Histone (H2A, H2B)*	*HTB2.1-HTA1HTB2.2-HTA2*	Ch8: 335-335 K Ch6: 643.4-645.6 K	Div.
*Tandem repeats*			
Old yellow enzyme*	*OYE2.5-OYE2.6-OYE2.8*	Ch4: 495.4-501.2 K	Tand., tand.
Old yellow enzyme	*OYE2.9-OYE2.4-OYE2.1*	Ch5: 313.2-317.5 K	Tand.
Aldo/keto reductase	*AKR1-AKR2*	Ch4: 949.8-952.4 K	Tand.
Aldo/keto reductase	*AKR3-AKR5*	Ch6: 51.7-54.2 K†	Tand.
Cinnamyl alcohol dehydrogenase	*CAD3-CAD2*	Ch1: 1857.3-1861.2 K	Tand.
2′ Hydroxyisoflavone reductase	*CIP1.1-CIP1.2-CIP1.3-CIP1.4-CIP1.5*	Ch5: 1530.2-1536.5 K	Tand.
Glutathione *S*-transferase	*GST1-GST3*	Ch2: 1967.5-19700 K	Tand.
Peptide transport	*PTR2.1-PTR2.2*	Ch2: 1024-1029 K	
Taurine catabolism	*IFH2.4-IFH2.3*	Ch1: 661-665 K	Tand.
Malate permease	*SSU1-SSU2-SSU3*	Ch3: 1225-1230.6 K	Tand.

* Also *Saccharomyces cerevisiae*.

† Subtelomeric.

Tand., tandem; div., divergent; conv., convergent.

Sequencing of the *P. stipitis* genome (Jeffries *et al.*, 2007) revealed the presence of at least 35 clusters of functionally related genes. Five occur in the subtelomeric regions of chromosomes (<30 kbp from the end). At least 18 clusters consist of two or more genes having different enzymatic activities that are functionally related. For example, maltose permeases are found in association with α-glucosidases; β-glucosidases are found in association with endoglucanases; and several genes for rhamnose metabolism, galactose metabolism, *N*-acetyl glucosamine degradation or pyrimidine metabolism are found adjacent to one another. In some instances, it is apparent that the pair or cluster formed, and then duplicated. In the case of the maltose permease/α-glucosidase pair, duplication of the gene pair was preserved.

Six other clusters consist of direct tandem repeats of divergent genes in ways similar to that reported in *S. cerevisiae* (Feuermann *et al.*, 1997). The gene clusters listed in Table 2 have been found by inspecting the genome; many more examples should be apparent from more exacting bioinformatic approaches.

In many cases, the clusters of genes having different activities but related functions are each themselves members of divergent orthologous families. For example *BGL, EGC*, *SUT, HXT* and maltose (*MAL*) (Jeffries, 2008) gene families are associated with one another in various combinations. By constructing dendograms of each of these gene families, it is possible to discern that in some instances (e.g. α-glucosidase/maltose permease and β-glucosidases/hexose transporter), the clusters appear to have formed and then the pairs of genes diverged together. In other instances, divergent members of these gene families are found unassociated with functionally related proteins (e.g. *BGL7, BGL4*, *EGC3* and *HXT2.1*).

**Fig. 2:**
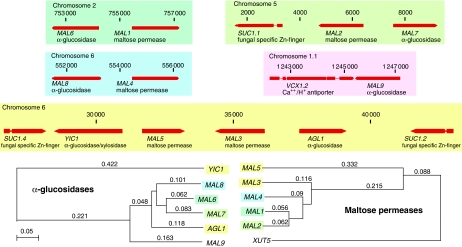
Evolutionary divergence of gene clusters for maltose transport and hydrolysis. Protein sequences for members of the α-glucosidase and maltose permease gene families were separately aligned using clustal w, and phylogenies were calculated using the best tree neighbor-joining and the Poisson correction. Physical clusters of the respective genes are shown in colored boxes. Other features are as per Fig. 1.

As noted in Table 2, any one pair of genes can be present in convergent, divergent or tandem orientations with respect to the direction of their transcription. The divergent orientation, in which two genes with related functions share a common 5′ sequence, has some implication with respect to regulation. This is the case with the α-glucosidase/maltose permease pairs. The orientation (and spacing) of the two genes has been maintained as the gene pair has duplicated. A comparison of the phylogenies for these two gene families gave rise to a series of diverging genes: *YIC1/MAL5*, ⇒*MAL9/MALx*⇒*AGL1/MAL3*⇒*MAL8/MAL4*⇒*MAL7/MAL2*⇒*MAL6/MAL1*. In the process, *MAL9* appears to have lost its associated permease and is now associated with a completely different transport protein (Fig. 2).

**Fig. 3:**
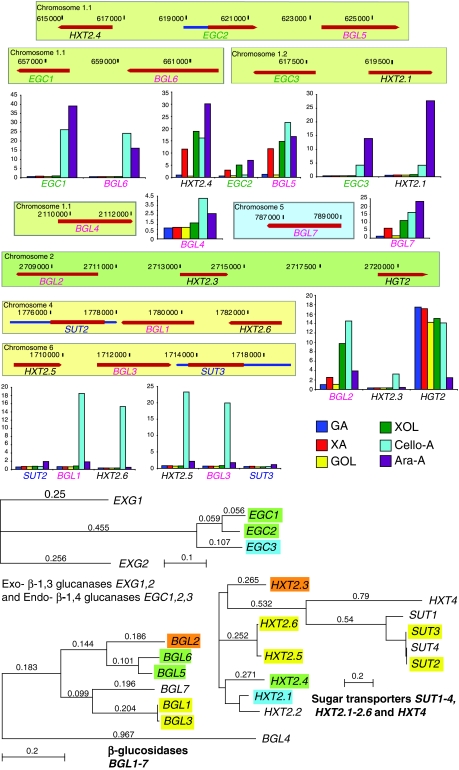
Correlation of gene clusters, phylogeny and expression for endo-glucanases, β-glucosidases and sugar transporters in *Pichia stipitis*. Each of the physical gene clusters are indicated by the colored blocks, and the phylogenetic relationships of the families are shown in the dendograms below. In addition to cultivation on glucose (G) or xylose (X) under aerobic (A) or oxygen-limited (OL) conditions, cells were cultivated in bioreactors on cellobiose (Cello) or l-arabinose (Ara) under aerobic conditions. Other features are as per Figs 1 and 2.

The regulatory profiles of gene clusters exhibit patterns that are characteristic of the genes with which they are associated and their evolutionary divergence. For example, on at least one occasion, a complete triplet cluster of β-glucosidases duplicated (cf. *BGL1/HXT2.6* and *BGL3/HXT2.5* clusters, Fig. 3). In this case, the transcriptional profiles of the two clusters are almost identical, with the genes strongly induced in the presence of cellobiose. Moreover, phylogenetic comparisons of the Hxt, Sut and Bgl protein families show that these protein pairs have barely diverged from one another (Fig. 3). A phylogenetic comparison of the seven putative β-glucosidases in *P. stipitis* shows that *BGL4* diverged earliest. This enzyme is most similar to fungal gentiobiases (β-1,6 glucosidase). It is expressed only at a very low level.

Phylogenetic comparisons of β-glucosidases associated with the endoglucanases show different regulatory profiles. The *EGC3/HXT2.1* pair might have diverged before its duplication and association with *BGL5*. The *EGC2/BGL5* part of the triplicate cluster then appears to have duplicated once again to form the *EGC1/BGL6* pair. The regulatory profile of *BGL7* is quite different from those of *BGL1* and *BGL3*, but quite similar to those of *BGL5* and *BGL2*. In the triplet cluster on chromosome 1, the *HXT2.4* and *BGL5* expression profiles are very similar, but *EGC2* is expressed to a lesser extent, suggesting that it might have been inserted between a progenitor of *BGL2/HXT2.3*. With the divergence of *EGC1/BGL6*, induced production of the endoglucanase in response to cellobiose and arabinose became much more pronounced, and the expression profile of *BGL6* evolved in a similar manner.

In a number of clusters, and most conspicuously in those that appear to have evolved in longest association with one another, the genes have a divergent orientation. Transcription proceeds in either direction from a central shared promoter sequence. This is true in all of the MAL gene clusters, several of the BGL gene clusters, the dityrosine cluster, the histone clusters and the GAL1-GAL10 cluster. Many of these highly evolved clusters are found over a wide taxonomic distance.

Examples of gene clusters are not confined to *P. stipitis*. Indeed, some of the most conspicuous clusters such as *DUR3-DUR1* (for urea metabolism), *GAL1-GAL10-GAL7* and the *MAL1* locus are found in *S. cerevisiae, D. hansenii* and other yeasts. Interestingly, the *P. stipitis* cluster contains a fourth gene, *GAL102*, which codes for a putative UDP-glucose 4-epimerase, along with a gene with unknown function (Table 2). Likewise, in comparison with the *MAL11, MAL12*, *MAL13* locus of *S. cerevisiae*, which contains the regulatory *GAL4*-like Zn-finger protein, *MAL13*, along with a maltose permease (*MAL11*) and an α-glucosidase (*MAL12*), the corresponding *P. stipitis* locus (Fig. 2) has two putative permeases and two highly divergent glucosidases – one of which (*YIC1*) is similar in structure to a xylosidase. Two fungal-specific Zn-finger proteins belonging to the same family as *GAL4* and *MAL13* flank these four genes in *P. stipitis*. Likewise, the *MAL2-MAL7* cluster has a flanking gene for a Zn-finger protein. The roles of these flanking Zn-finger genes and their associated proteins in regulation are unclear.

The dityrosine metabolic cluster, which codes for genes related to spore formation, is coregulated, as one might expect. These are coregulated with *AUT4* for autophagy, which also happens to be colocated in the genome, along with a chitinase, which would function to hydrolyze the vegetative cell wall. These genes are downregulated on cellobiose aerobic or xylose under oxygen limitation. The *NAG1, 2* and *4* genes likewise show similar regulation properties.

Table 2 is not exhaustive and consists principally of gene clusters that have been recognized in the course of annotating the genome. Indeed, Watanabe *et al.* (2008a,b); discovered a cluster of four genes for an alternative l-rhamnose metabolic pathway, even though several of the genes in the cluster did not have a known function before their study (Koivistoinen *et al.*, 2008). Bioinformatic studies combining genomic and expression array data will probably reveal more unknown metabolic associations and pathways.

Clearly, much more can be learned about regulation of genes relevant to lignocellulose bioconversion through the comparative study of gene clusters in different yeasts. Gene clusters often appear to be coregulated with genes in each cluster showing similar induction patterns. Gene clusters such as *MAL6* and *MAL1*, which appear to be highly specific for maltose utilization, are highly induced when cells are grown on maltose, while other clusters show broader patterns of induction. A more comprehensive comparison of *MAL, GAL* and *DUR* clusters across taxonomic lines could provide a better picture of how these traits have evolved in yeasts.

## Tandem duplication

Tandem duplication appears to be an early stage in the formation of gene families. It is not entirely clear how new genes either enter the genome or become amplified. Some appear to be acquired through lateral transfer from other yeasts, other fungi or bacteria.

The *OYE* gene family, which codes for NADPH dehydrogenase, is present in 12 members, six of which appear in two triplet clusters of tandem repeats. *OYE* genes are present in other related genomes. *Debaryomyces hansenii* has an even larger cluster of OYE genes, five in all, that are found on Chromosome C (Dujon *et al.*, 2004). Three of these are very similar to PsOYE2.5, 2.5 and 2.8 on PsCh 4. These clusters correspond to a similar OYE cluster in *Pichia guilliermondii* (PGUG_05760-PGUG_05763) (Broad Institute 2009), which is flanked by putative NAD/NADP-linked alcohol dehydrogenases proximal to either end (PGUG_05764 and PGUG_05758). A similar ADH (*PsADH5*) is proximal to the triplet OYE cluster in *P. stipitis*. Phylogenetic analysis of the *P. stipitis* OYE genes suggests that a triplicate cluster underwent three rounds of duplication, followed by differentiation with little or no loss of the intermediate activities. Regulatory profiles of the tandem genes show little similarity except among genes that have recently duplicated. The proliferation and differentiation of these genes probably represents the fulfillment of new metabolic niches. *Pichia stipitis* occurs in both free-living and insect-associated forms, and the different NADH dehydrogenases would be required for these adaptations.

Likewise, the AKR family of aldo/keto reductases – to which *XYL1* for xylose reductase belongs – is present in six members with two paired clusters. Survival of duplicated pairs and triplicates in the genome implies that higher levels of these enzymatic activities were required for survival of the yeast in its habitat.

Cinnamyl alcohol reductases (*CAD*) are present in five copies, of which two (*CAD2/CAD3)* exist in a tandem repeat. *CAD5* is the most divergent. Very few examples can be found in related genomes for this enzymatic activity. This, therefore, represents an idiomatic activity. Tandem duplication seems to be characteristic of newly acquired genes for which expression levels are low. Following duplication, proteins stemming from a common function could evolve into enzymes with different kinetic properties that would improve cellular metabolism. Speculation about the functions of these multiple genes is not really warranted in the absence of additional biochemical, physiological study, but the emergence, proliferation and divergence of such clusters into gene families are certainly of interest.

**Fig. 4:**
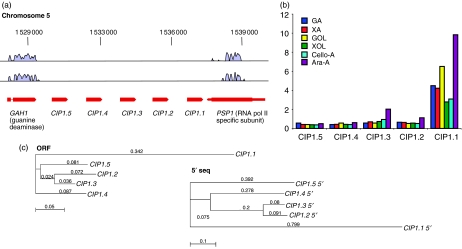
*CIP1* gene cluster. (a) Genomic organization of the cluster. (b) Gene expression data. Triplicate cultures were grown at 30°C, pH 5, in 2 L of minimal defined medium with 50 g L^−1^ glucose (G), xylose (X), cellobiose (Cello) or arabinose (Ara) under fully aerobic (A) or oxygen-limited (OL) conditions. Cell samples were harvested for mRNA, converted to cDNA and hybridized against 60-mer NimbleGen expression arrays. The average relative expression levels of normalized triplicate samples are shown. (c) Different rates of evolutionary divergence for *CIP1* genes in a tandemly duplicated cluster. Protein sequences and the 5′ sequences were separately aligned using clustal w and phylogenies were calculated using the best tree neighbor-joining and the Poisson correction.

The largest gene cluster of tandem repeats thus far recognized in *P. stipitis* is a set of five genes coding for apparent 2′ hydroxyisoflavone reductases (*CIP1.1-1.5*). Homologs with weak similarity are found in two copies in *D. hansenii* and *Yarrowia lipolytica*, but the cluster of *CIP1* genes appears to be idiomatic to *P. stipitis*. The function of this enzymatic activity in *P. stipitis* is unknown. Four out of five gene copies are transcribed minimally under all of the growth conditions tested. One copy, however, *CIP1.1*, is expressed at significant levels and has evolved through selection much more than the others. Because the five direct repeats are unusual in their repetitive nature, it is possible to trace the evolutionary divergence of the 5′ sequences in addition to the ORFs. As can be seen in Fig. 4, the evolutionary divergence of the promoter sequences parallel those of the structural genes, but at about twice the rate. The promoter of *CIP1.1* apparently acquired several 12–24-bp segments not found in the other sequences. These presumably increased the expression and allowed selection to work more effectively on this paralog.

## Resequencing – what we learned about selection

Direct and complete resequencing of the *P. stipitis* genome after several rounds of mutagenesis, selection and cultivation over several years has provided an insight into how adaptation occurs at a molecular level. Surprisingly, only 14 point mutations were preserved during this process (Smith *et al.*, 2008). Of these, 10 were in ORFs and four were in intergenic regions.

All of the mutations in ORFs resulted in amino acid substitutions, and seven of these resulted in significant changes in the amino acid function. The degeneracy of the genetic code often preserves the same amino acid residue when mutations occur in the third position, and the structure of the code tends to conserve hydrophobic, hydrophilic, acidic, basic and other amino acid characteristics even when substitutions occur; hence, the prevalence of mutations altering amino acid function has a very low probability of being attributable to random events.

Selection for resistance to inhibitors, growth on limiting carbon sources and selection for more rapid growth or fermentation was used at each stage in the selection process; hence, the resulting mutations probably provided adaptive advantages for growth under the selective conditions used. Unfortunately, our limited knowledge of the functions of the affected genes does not permit a rational interpretation of the genotypes.

## Conclusions

Similar gene clusters and regulatory patterns could probably be observed in many different yeasts. Their recognition in *P. stipitis* is the result of concerted annotation and expression array studies. What we can see from this, however, is that gene families appear to arise from acquisition, duplication and differentiation within a genome. When complementary activities become colocated through the process of resortment, evolution of the cluster can proceed through duplication and further codifferentiation. *Cis*-acting regulatory features can evolve more rapidly and can be coordinated across several genes in a cluster. In this process, a divergent gene orientation with common 5′ sequences tends to be preserved. Concerted studies of structure, function and regulation in yeast genomes are likely to result in many more insights into cell physiology and evolution.

## Statement

Reuse of this article is permitted in accordance with the Creative Commons Deed, Attribution 2.5, which does not permit commercial exploitation.
